# Medication and Dietary Supplement Interactions among a Low-Income, Hospitalized Patient Population Who Take Cardiac Medications

**DOI:** 10.1155/2015/429826

**Published:** 2015-04-12

**Authors:** Paula Gardiner, Amanda C. Filippelli, Ekaterina Sadikova, Laura F. White, Brian W. Jack

**Affiliations:** ^1^Department of Family Medicine, Boston University School of Medicine, Boston Medical Center, Boston, MA 02118, USA; ^2^Department of Biostatistics, Boston University School of Public Health, Boston, MA 02118, USA

## Abstract

*Purpose*. To identify characteristics associated with the use of potentially harmful combinations of dietary supplements (DS) and cardiac prescription medications in an urban, underserved, inpatient population. *Methods*. Cardiac prescription medication users were identified to assess the prevalence and risk factors of potentially harmful dietary supplement-prescription medication interactions (PHDS-PMI). We examined sociodemographic and clinical characteristics for crude (*χ* ^2^ or *t*-tests) and adjusted multivariable logistic regression associations with the outcome. *Results*. Among 558 patients, there were 121 who also used a DS. Of the 110 participants having a PHDS-PMI, 25% were asked about their DS use at admission, 75% had documentation of DS in their chart, and 21% reported the intention to continue DS use after discharge. A multivariable logistic regression model noted that for every additional medication or DS taken the odds of having a PHDS-PMI increase and that those with a high school education are significantly less likely to have a PHDS-PMI than those with a college education. *Conclusion*. Inpatients at an urban safety net hospital taking a combination of cardiac prescription medications and DS are at a high risk of harmful supplement-drug interactions. Providers must ask about DS use and should consider the potential for interactions when having patient discussions about cardiac medications and DS.

## 1. Introduction

A dietary supplement (DS) is intended to supplement the diet and contains one or more dietary ingredients such as vitamins, minerals, herbs or other botanicals, amino acids, and other substances or their constituents [1]. DS use remains prevalent in a variety of patient populations including those who are frequently hospitalized, prescription medication users, those with chronic health conditions, and the elderly [2–5]. DS are also commonly used by patients for cardiovascular health [6]. A systematic review of patients with cardiovascular disease found that the prevalence of biologically based therapies ranged from 22% to 68% [7]. This paper explores cardiac medication and DS use among inpatients.

Many patients take DS to promote health and prevent complications from cardiac health conditions. In a study examining patients enrolled in a cardiac rehabilitation program, 67% of patients enrolled were taking vitamins, with or without minerals, with multivitamins and fish oil being the most common supplements used [8]. There is extensive research identifying DS with cardiovascular effects. Research studies show that DS carry antiplatelet, antiarrhythmic, and antihyperlipidemic properties that can be beneficial for some cardiac patients [9, 10]. Fish oil, for example, has been shown to reduce triglycerides and improve vascular health [11]. Despite the popular use of DS, there is conflicting evidence over the efficacy and safety of some DS in patients with cardiac disease, especially patients in inpatient hospitalized settings [12–14].

While much is known about the risks, benefits, or potential interactions (synergistic or antagonistic) between cardiac medications and DS, little is known about how these risks, benefits, or interactions impact hospitalized patients [13, 15]. For example, some DS-medication combinations carry an increased risk of bleeding, particularly when antiplatelet or anticoagulant therapy is involved [16]. Hospitalized patient populations may be more susceptible to DS-medication interactions due to their acute or chronic illness or inpatient treatment plans (e.g., surgery, intravenous anticoagulation). If providers are unaware of a patient's home DS use or if medical records lack proper documentation of DS use, providers may unknowingly prescribe a treatment regimen or a discharge medication that could harm a patient [17].

We performed an analysis of a cohort of hospitalized low-income, racially diverse prescription medication users enrolled in the Re-Engineered Discharge (RED) clinical trials to determine the prevalence of potentially harmful dietary supplement-prescription medication interactions (PHDS-PMI) [18]. We aimed to identify patient characteristics that are associated with the use of DS in an ethnically diverse, inpatient population. Previous studies have shown a trend that patients with multipharmacy are more likely to have DS interactions [19]. We wanted to see if this trend is also true in low-income, racially diverse patients. Thus, we hypothesized that patients who take multiple products are more likely to have a PHDS-PMI.

In this paper, we explore inpatient characteristics associated with patients' use of potentially harmful combinations of DS and prescription cardiac medications. We also identify classes of cardiac medications used by patients with PHDS-PMI. Finally, we address the occurrence of patient-clinician communication about DS use during the studied admission.

## 2. Methods

### 2.1. Sample Population

The study sample consisted of participants from the Re-Engineered Discharge (RED) clinical trials conducted at Boston Medical Center (BMC), in Boston, Massachusetts [18]. The RED trials were conducted between 2009 and 2010 to test a newly designed discharge process with inpatients at BMC. Patients 18 years of age and older with the ability to speak English were enrolled in the studies (*N* = 802). Patients were excluded if they were admitted to BMC from a skilled nursing facility or other hospital, were admitted for a planned hospitalization, were on hospital precautions or on suicide watch, or were deaf or blind.

The sample used in the current analyses was restricted to RED participants who used a cardiac medication and were administered questions about any DS use and communication with health care professionals at their baseline interview by a trained research assistant (*N* = 623). Participants were asked about their use of herbal supplements, vitamins, minerals, or nonvitamin/mineral supplements in the last 12 months, and specifically about the use of fish oil/omega fatty acids, cranberry, garlic, ginseng, ginger, gingko biloba, St. John's Wort, or other supplements (an open ended question). From this subsample (*n* = 623), we conducted an inpatient chart review to identify patients who had at least one prescription medication listed on their admission history and physical (*n* = 558). We extracted all the cardiac medications from the admission medication list, categorizing them into antihypertensives, antiarrhythmics, anticoagulants, cholesterol-lowering, and diabetes medications. Antiplatelet medications were included in the anticoagulant category. Medications were categorized as per the classification of Micromedex, a point of care medication management database [20]. We extracted any DS including vitamins/minerals such as vitamin C, vitamin D, multivitamins, calcium, magnesium, herbal, or other nonvitamin/mineral supplements (e.g., fish oil). We included diabetes medications in our analysis as many patients with diabetes have cardiac disease and it is routine to prescribe angiotensin converting enzyme (ACE) inhibitors [21].

Finally, participants were asked about their communication with the inpatient team during the baseline interview. The following questions were asked: “During your current stay, did a doctor or nurse ASK YOU about your use of vitamin, minerals, and herbs?” and “Will you continue to use your vitamins, minerals, or herbs when you go home from the hospital?” This study was approved by the Boston Medical Center Institutional Review Board.

### 2.2. Sample Characteristics

Clinical and sociodemographic variables included age (continuous variable), gender, education level (less than high school, high school or equivalent, or at least some college), income (unknown/refused, none–$9,999, $10,000–$29,999, $30,000–$49,999, or $50,000+), insurance (private or government/free), race (Non-Hispanic Black, Non-Hispanic White, or Hispanic/other (Asian/Pacific Islanders, American Indians)), and marital status (single or married). English as a primary language and being born in the US were dichotomized (yes/no). Health literacy was measured using the Rapid Estimate of Adult Literacy in Medicine (REALM) scale [22]. As it has been done by previous authors, REALM was categorized as low health literacy for subjects with a REALM score less than 60 (8th grade and below) and higher health literacy for subjects with a REALM score greater than or equal to 60 (9th grade and above) [23–25]. Depressive symptoms were measured by the Patient Health Questionnaire (PHQ-9) and divided into two categories: no depressive symptoms (PHQ score < 5) or mild to severe depressive symptoms (PHQ score ≥ 5) [26].

To asses for health care utilization, we looked at the frequency of emergency room (ER) use or inpatient hospitalization (2 or more hospitalizations or ER visits in the 6-month period prior to enrollment) and whether the patient had a primary care physician (PCP).

To assess for lifestyle variables, we looked at alcohol use in male participants with the question “Have you had 5 or more drinks in one sitting in the past year?” (4 or more drinks for female participants). Answers were dichotomized (yes/no) for analysis. Illegal drug use was assessed with the question “Have you used any illegal drugs or prescription drugs for a nonmedical use?” and was dichotomized (yes/no) for analysis.

Finally, we created a continuous variable which represented a count of the total number of medications and DS the patient was reportedly using upon admission (the number of medications or supplements documented in the chart). We will refer to this variable as the number of oral products throughout the paper.

### 2.3. Outcome Assessment

The primary outcome of the analysis was the presence of a PHDS-PMI based on self-reported or documented DS and medications in the admission note (yes/no). We defined a PHDS-PMI to be the concomitant use of a DS and a cardiac medication (antihypertensives, antiarrhythmics, anticoagulants, cholesterol-lowering medications, and diabetes medications) that was rated to have* moderate or major* severity by the Natural Medicines Comprehensive Database [27]. A moderate interaction advises “Use cautiously or avoid combination; warn patients that a significant interaction or adverse outcome could occur” [27]. A major interaction advises “Do not use combination; contraindicated; strongly discourage patients from using this combination; a serious adverse outcome could occur” [27]. A participant was considered to have the primary outcome if he or she had at least one PHDS-PMI.

### 2.4. Statistical Analysis

Sociodemographic and clinical characteristics were compared between those with at least one PHDS-PMI and those without using chi-square tests for dichotomous and categorical variables and *t*-tests for continuous variables. A manual stepwise selection was performed to include variables into a logistic regression, using entrance and exit criteria of a *P* value <0.2. Age and gender were included into the model for adjustment. Due to missing data, the final sample size used for the logistic regression was 546 participants. All statistical analyses were performed with SAS Version 9.3.

## 3. Results

Of the full sample of 802 RED participants, 623 were administered the DS questions. Compared to the 179 RED participants who were not included in our analyses, those who were included had significantly lower hospitalization use and higher health literacy but did not differ on other sociodemographic, health behavior, or diagnosis-related characteristics. Of the 623 participants, 558 had at least one cardiac related prescription medication (antihypertensives, antiarrhythmics, anticoagulants, cholesterol-lowering, and diabetes medications) on their admission history and physical.

Of the full sample of participants who had documented use of at least one cardiac prescription medication (*n* = 558), 131 (23.5%) had 1–3 prescription medications, 186 (33.3%) had 4–7, and 115 (20.6%) had 8–11, while 126 (22.6%) had 12 or more prescription medications listed. The maximum number of prescription medications recorded was 28.  Table 1 reports the sociodemographic factors associated with and without potentially harmful dietary supplement-prescription medication use. Of 558 cardiac prescription medication users, 121 individuals in our sample had both DS and prescription medications documented in their history and physical. With the change to bold numbers, the sentence should read. Out of the 121, 110 (90.9%) had at least one PHDS-PMI (interactions are denoted with bold numbers in Table 2).

For those patients who took antihypertensive medications (*n* = 252), 173 (68.7%) also took at least one DS. Of those 173 patients, 51.4% were at risk for a PHDS-PMI. For those patients who took antiarrhythmics or nitrate medications (*n* = 95), 16 (16.8%) also took at least one DS. Of those 16 patients, 75% had a PHDS-PMI. For those who took anticoagulants (*n* = 186), 107 (57.5%) also took at least one DS. Of those taking an anticoagulant and DS (*n* = 107), 57.9% had a PHDS-PMI. For patients taking cholesterol-lowering medications (*n* = 178), 99 (55.6%) also took a DS. Of those 99 patients, 57.6% had a PHDS-PMI.

Table 3 summarizes inpatient behaviors such as being asked about DS use, having documentation in the chart of DS use, and if they will continue to use DS when discharged (% among those identified with PHDS-PMI). Twenty-five percent of any cardiac medication users with PHDS-PMI were asked about their DS use by the inpatient team at admission, and 21% planned to continue use of DS after discharge. Participants on Warfarin and other anticoagulant medications and who reported a concomitant DS to use after discharge reported very low rates clinical inquiry. None of the Warfarin users with a concomitant DS were asked about DS use, while 9% of other anticoagulant medication users, reporting a DS, reported being asked about DS use.

Unadjusted comparisons of baseline participant data indicate that participants with PHDS-PMI were more likely to use a higher number of products (*P* = 0.004). An adjusted logistic regression analysis of the odds of having a PHDS-PMI reveals that taking an additional medication or supplement significantly increases the odds of having a drug-DS interaction (OR: 1.07; 95% CI: 1.03, 1.11), while having a high school education significantly decreases the odds for having a PHDS-PMI than those with a college education (OR: 0.47; 95% CI: 0.28, 0.79). There were no statistically significant differences between any levels of household income compared to the lowest household income level (Table 4).

## 4. Discussion

Our analysis brings new findings about the interactions of DS and prescription medications in a low-income, hospitalized population. Among 558 inpatients, 121 participants taking a prescription cardiac medication also used a DS. Of the 110 participants having a PHDS-PMI, 25% were asked about their DS use at admission, 75% had documentation of DS in their chart, and 21% reported the intention to continue DS use after discharge. We found that taking an additional medication or DS significantly increases the odds of having a PHDS-PMI, while having at least some college education is associated with having a greater chance of an interaction.

An analysis of the National Health and Nutrition Examination Survey (NHANES) from 2005 to 2008 found that one-third of all US adults reported concomitant use of DS and prescription medications, with the most common prescription medications used with DS being cardiovascular medications [28]. An analysis of the NHANES from 1999 to 2002 found that persons with coronary artery disease/stroke used more DS, specifically vitamin E, folic acid, niacin, and fish oil [29]. In our study, we also found that patients frequently reported using fish oil/omega fatty acids (*n* = 60). Other frequently used supplements among our population were multivitamins (*n* = 56), cranberry supplements (*n* = 54), garlic supplements (*n* = 46), and calcium (*n* = 36).

DS are associated with promoting cardiovascular health [30]. In a study of patients with heart failure, participants reported that DS were important for overall health and almost all informed their physician of their DS use [31]. In contrast, a different study examining patients with heart failure found that 35% took herbal supplements, and 65% took vitamins [32]. Yet, the majority of these study participants did not report this usage to health care providers. As patients with cardiovascular disease engage in health-promoting activities, clinicians need to be aware that DS use is often associated with positive lifestyle choices [33].

We found that the fact that participants take an additional medication or supplement significantly increases the odds of having a drug-DS interaction. Our results mirror those of others who found that the odds of an exposure to a potential medication interaction were increased with the number of products used [19]. Since many cardiac patients are often on several medications, they are at increased risk of interactions [13, 16, 34, 35]. One cross-sectional study found that the most common herb-drug combination was between bilberry and antihypertensives, analgesics, or anticoagulants [36]. Due to the complexity of medication regimens, cardiac patients must have careful and consistent medication reconciliation to ensure that they are on safe medication and DS combinations.

In our analyses, we found that having a high school education significantly decreases the odds for having a PHDS-PMI than those with a college education. This is in contrast to previous research that has shown that higher education is associated with concomitant DS and prescription medication [28]. While we did not find any association between patient income and risk to PHDS-PMI, a recent study found that those with higher income were more likely to consume conventional medications and Chinese herbal products [37]. Future research is necessary to examine how a patient's level of education and income influence whether or not a patient is at risk to PHDS-PMI.

Documentation of DS allows for coordinated, comprehensive care for patients who are managed by several specialists. In our study of participants having a PHDS-PMI, 25% were asked about their DS use at admission and 75% had documentation of DS in the medical chart. In a chart review of patients in a cardiac rehabilitation program, 67% had documentation of DS with multivitamins and fish oil being the most common [8]. Without proper discussion and documentation of medications and DS, providers run the risk of making a medical error. One study found that cardiovascular medications were involved in 41% of all medication errors on cardiology wards [38]. Proper documentation of DS is critical to ensuring continuity of care from one provider to the next. More research is needed to assess how many adverse reactions are attributed to failure to ask about and document DS.

Our study has several limitations. First, participants were exclusively English-speaking patients. This limits our ability to get information from non-English-speaking patients about their DS use. Another limitation is that admission note prescription medication lists may be incomplete. We were also unable to assess whether clinicians had undocumented conversations about DS use with patients that the patients did not recall. In addition, since this research was conducted in an urban, underserved medical center, the findings may not be generalizable to other patient populations. It is also unknown if drug-DS interactions negatively affect the long term health of cardiac patients or if this concurrent use has hidden benefits to chronically ill patients.

In conclusion, drug-DS interactions commonly occur among patients who take cardiac medications. Cardiac patients may be especially at risk to adverse health events, as cardiac medications such as Warfarin and antiarrhythmics have small therapeutic thresholds for dosing. It is important for providers to inquire about DS use during transitions of care and clinical encounters to ensure that patients are not at risk to PHDS-PMI. By facilitating open conversations, clinicians can prescribe safe therapeutic regimens.

## Figures and Tables

**Table 1:** Sociodemographic factors associated with and without potentially harmful dietary supplement-prescription medication use.

Sociodemographic factors	Total number of participants	Potentially harmful dietary supplement-prescription medication use		*P* value
Sample size	(*N* = 558)	No (*N* = 448)	Yes (*N* = 110)	
Continuous age: mean (SD)	558	49.86 (13.75)	49.52 (16.25)	0.84
Number of oral products: mean (SD)	558	7.89 (5.23)	9.55 (5.62)	0.004
Gender				
Male	276	225 (81.52%)	51 (18.48%)	0.47
Female	282	223 (79.08%)	59 (20.92%)	
Education^*^				
Less than high school	112	85 (75.89%)	27 (24.11%)	0.05
High school	227	195 (85.9%)	32 (14.1%)	
College	207	158 (76.33%)	49 (23.67%)	
Income				
Other (refused/missing)	206	170 (82.52%)	36 (17.48%)	0.17
None to $9,999	124	92 (74.19%)	32 (25.81%)	
$10,000–$29,999	121	97 (80.17%)	24 (19.83%)	
$30,000–$49,999	48	43 (89.58%)	5 (10.42%)	
$50,000+	59	46 (77.97%)	13 (22.03%)	
Insurance				
Private	184	145 (78.8%)	39 (21.2%)	0.54
Government/free care	374	303 (81.02%)	71 (18.98%)	
Race				
Non-Hispanic Black	278	221 (79.5%)	57 (20.5%)	0.67
Hispanic/other	108	90 (83.33%)	18 (16.67%)	
Non-Hispanic White	172	137 (79.65%)	35 (20.35%)	
Marital status				
Single	436	350 (80.28%)	86 (19.72%)	0.99
Married	122	98 (80.33%)	24 (19.67%)	
English as the primary language				
Yes	488	394 (80.74%)	94 (19.26%)	0.48
No	70	54 (77.14%)	16 (22.86%)	
Born in the USA				
Yes	434	350 (80.65%)	84 (19.35%)	0.69
No	124	98 (79.03%)	26 (20.97%)	
REALM^**^				
≤60	205	167 (81.46%)	38 (18.54%)	0.59
>60	328	261 (79.57%)	67 (20.43%)	
Depressive symptoms				
Mild to severe	92	76 (82.61%)	16 (17.39%)	0.54
No	466	372 (79.83%)	94 (20.17%)	
Health care utilization				
Frequent utilizer				
Yes	85	70 (82.35%)	15 (17.65%)	0.60
No	473	378 (79.92%)	95 (20.08%)	
Has PCP				
Yes	455	360 (79.12%)	95 (20.88%)	0.15
No	103	88 (85.44%)	15 (14.56%)	
Lifestyle variables				
Alcohol use				
Yes	146	116 (79.45%)	30 (20.55%)	0.77
No	412	332 (80.58%)	80 (19.42%)	
Illegal drug use				
Yes	100	86 (86.0%)	14 (14.0%)	0.11
No	458	362 (79.04%)	96 (20.96%)	

^*^Total participants = 546.

^**^Total participants = 533.

**Table 2:** Specific medication and dietary supplement concordant use self-reported or documented in the medical record.

	High blood pressure medication				Antiarrhythmic and nitrates	Anticoagulants except Warfarin	Warfarin	Cholesterol-lowering drugs (statins and fibrates)	Diabetes medications
Total *N* = 558	Diuretics *N* = 171	ACE inhibitors *N* = 162	ARBs *N* = 99	Other *N* = 198	*N* = 95	*N* = 175	*N* = 20	*N* = 178	*N* = 152
Any vitamins/minerals^‡^ (*N* = 296)	81	73	46	90	48	79	8	80	73
Vitamin C^*^ (*N* = 6)	1	1	0	**2**	0	2	0	**1**	1
Vitamin D^*^ (*N* = 46)	18	17	4	**23**	**4**	**17**	**2**	**21**	18
Multivitamin^*^ (*N* = 56)	16	20	3	**24**	2	**18**	**5**	**18**	**16**
Calcium^*^ (*N* = 36)	**14**	11	4	**13**	**1**	13	3	**12**	11
Magnesium^*^ (*N* = 4)	**2**	0	1	**2**	1	2	1	0	2

Any nonvitamin dietary supplement^‡^ (*N* = 169)	35	35	14	47	10	39	6	39	30
Fish oils/omega fatty acids^‡^ (*N* = 60)	**14**	**10**	**6**	**16**	5	**16**	**2**	16	15
Cranberry supplement^‡^ (*N* = 54)	11	11	7	15	3	19	**2**	18	13

Garlic supplements^‡^ (*N* = 46)	9	9	**5**	**12**	2	**13**	**1**	12	8
Ginseng supplement^‡^ (*N* = 18)	5	6	1	**6**	0	**7**	**2**	**8**	**7**
Ginger supplements^‡^ (*N* = 26)	4	4	2	5	0	**7**	**1**	6	6
Gingko biloba supplement^‡^ (*N* = 3)	2	1	**1**	**2**	**0**	**2**	**1**	2	**2**
St. John's Wort^‡^ (*N* = 2)	0	0	**0**	**0**	**0**	0	**0**	**0**	0

Interaction rates by drug class	89/252 = 0.29				12/95 = 0.04	56/186 = 0.31		57/178 = 0.24	46/152 = 0.15

^*^Documented in chart.

^‡^Self-reported.

ACE: angiotensin converting enzyme inhibitors.

ARB: angiotensin II receptor blockers.

Other blood pressure medications (beta blockers, calcium channel blockers, and prostaglandins).

**Table 3:** Behaviors surrounding DS use among participants with drug-specific interactions.

Type of cardiac medication	*N* with DS interaction	Asked	Documented	Continue
Any cardiac medication	110	27 (25%)	83 (75%)	23 (21%)
Hypertension medication	89	8 (9%)	69 (78%)	24 (27%)
Antiarrhythmic medication	12	0 (0%)	7 (58%)	5 (42%)
Anticoagulants (except Warfarin)	56	5 (9%)	39 (44%)	16 (18%)
Warfarin	10	0 (0%)	9 (90%)	3 (30%)
Cholesterol-lowering medication	57	5 (9%)	40 (70%)	16 (28%)
Diabetes medication	46	3 (6%)	34 (74%)	12 (26%)

“Cardiac medications” is a compilation of all of the following (see Table 2 for individual medication frequencies).

**Table 4:** Adjusted multivariable logistic regression reporting factors associated with participants with and without potentially harmful dietary supplement-prescription medication use^*^.

Number of observations used: 524 participant characteristics	Odds ratio (95% CI)	*P* value
Number of oral products	1.07 (1.03, 1.11)	0.0009
Annual household income		
$10,000–$29,999 versus none to $9,999	0.70 (0.38, 1.29)	0.33
$30,000–$49,999 versus none to $9,999	0.30 (0.10, 0.76)	0.09
$50,000+ versus none to $9,999	0.49 (0.20, 1.10)	0.61
Missing/refused versus none to $9,999	0.58 (0.33, 0.998)	0.94
Education		
High school versus college	0.47 (0.28, 0.79)	0.0015
Less than high school versus college	1.00 (0.57, 1.76)	0.14
Insurance		
Government/free care versus private	0.82 (0.51, 1.34)	0.42
Illegal drug use		
Yes versus no	0.59 (0.31, 1.07)	0.098

^*^Adjusted model for age and gender.
